# Biodetection of a specific odor signature in mallard feces associated with infection by low pathogenic avian influenza A virus

**DOI:** 10.1371/journal.pone.0251841

**Published:** 2021-05-26

**Authors:** Glen J. Golden, Meredith J. Grady, Hailey E. McLean, Susan A. Shriner, Airn Hartwig, Richard A. Bowen, Bruce A. Kimball

**Affiliations:** 1 Department of Biomedical Sciences, Colorado State University, Fort Collins, CO, United States of America; 2 USDA-APHIS-WS-National Wildlife Research Center, Fort Collins, CO, United States of America; 3 Monell Chemical Senses Center, Philadelphia, PA, United States of America; Radboud Universiteit, NETHERLANDS

## Abstract

Outbreaks of avian influenza virus (AIV) infection included the spread of highly pathogenic AIV in commercial poultry and backyard flocks in the spring of 2015. This resulted in estimated losses of more than $8.5 million from federal government expenditures, $1.6 billion from direct losses to produces arising from destroyed turkey and chicken egg production, and economy-wide indirect costs of $3.3 billion from impacts on retailers and the food service industries. Additionally, these outbreaks resulted in the death or depopulation of nearly 50 million domestic birds. Domesticated male ferrets (*Mustela putorius furo*) were trained to display a specific conditioned behavior (i.e. active scratch alert) in response to feces from AIV-infected mallards in comparison to feces from healthy ducks. In order to establish that ferrets were identifying samples based on odors associated with infection, additional experiments controlled for potentially confounding effects, such as: individual duck identity, housing and feed, inoculation concentration, and day of sample collection (post-infection). A final experiment revealed that trained ferrets could detect AIV infection status even in the presence of samples from mallards inoculated with Newcastle disease virus or infectious laryngotracheitis virus. These results indicate that mammalian biodetectors are capable of discriminating the specific odors emitted from the feces of non-infected versus AIV infected mallards, suggesting that the health status of waterfowl can be evaluated non-invasively for AIV infection via monitoring of volatile fecal metabolites. Furthermore, in situ monitoring using trained biodetectors may be an effective tool for assessing population health.

## Introduction

The idea that changes in body odor alterations can be diagnostic for disease diagnosis has been around since the time of Hippocrates (around 400 bce). Extending this concept to the surveillance of disease in wildlife populations is relatively new in comparison but is gaining support as more evidence is presented that certain infectious diseases can alter human and animal body odors. Efforts to exploit this phenomenon have increased, with multiple demonstrations that such biodetectors are accurate and efficient in the detection and surveillance of disease [1, 2].

Due to devastating losses of farmed fowl, AIV has been identified for its potential to disrupt the economy of the poultry industry [3, 4]. Waterfowl and shorebirds are the natural reservoir of all avian subtypes of AIV, distributed across the globe, and considered primarily responsible for the spread and maintenance of AIV in nature [3, 4]. Wild waterfowl and shorebirds do not typically exhibit clinical signs of infection in nature [3–5]. Furthermore, there is evidence that highly pathogenic (HP) AIV strains may follow introduction of low pathogenic (LP) AIV by wild birds and subsequently mutate within poultry [6–8]. Given the potential impacts of AIV to domestic animals and human health, it is imperative that new, reasonably cost-effective tools be developed for detection of AIV infection.

In a preliminary study, we demonstrated that ferrets could be trained to display a specific conditioned behavior (i.e. active scratch alert) in response to a marked increase of acetoin when presenting varying ratios of acetoin (3-hydroxy-2-butanone) and 1-octen-3-ol which were diagnostic of AIV infection. After successfully completing this discrimination task, ferrets rapidly generalized this learned response to the odor of feces from AIV- infected mallards. This confirmed earlier research using trained mice to discriminate infection status of ducks on the basis of fecal odors [9]. In this same study, chemical analyses indicated that AIV infection was associated with a marked increase of acetoin, previously identified as a biomarker for diagnosing gastrointestinal diseases in humans [10], and was the impetus for initially training the ferrets to varying acetoin concentrations.

We hypothesized that successful AIV detection by trained mice could be repeated in a species that had a more malleable behavioral repertoire, specifically dogs. Toward our plans for developing a viable biodetector program, ferrets were first chosen as a “bridge” to evaluate the concept. Ferrets were chosen on evidence that domestic ferrets readily learn discrimination tasks and exhibit dog-like social-cognitive skills when interacting with humans [11, 12] and a previous study that showed ferrets could discriminate odors by detecting peppermint odor [13].

In the current study, domesticated male ferrets (*Mustela putorius furo*) were trained to display a specific conditioned behavior (i.e., active scratch alert) in response to feces from mallards experimentally infected with low pathogenic AIV. Ferrets were able to discriminate samples from infected versus noninfected ducks in several experiments. Bioassays were performed to control for potentially confounding effects such as individual duck identity, virus infection dose, and infection day (post-treatment day of fecal sample collection). Furthermore, we evaluated if ferrets generalized the learned LPAIV response to other viruses. This test of specificity was a critical evaluation of the utility of trained biodetectors for field evaluation.

## Materials and methods

### Ethics statement

The experimental protocol for ferret research were approved by the National Wildlife Research Center (QA-2504) and the protocols for experimental infection were approved by the National Wildlife Research Center (QA-1912) and the Colorado State University (17-7544A) Institutional Animal Care and Use Committees.

### Biodetectors

Six castrated male ferrets were acquired at 15 weeks of age from Marshall BioResources (North Rose, NY) and trained as described in a previous study (PONE-D-20-11704R1). Ferrets were pair-housed at the National Wildlife Research Center (Fort Collins, CO) in two level wire cages (MidWest, Muncie, IN) and maintained at 23°C on a 12 hour light (12 hour dark) cycle. The ramp connecting the upper and lower levels of the cage could be locked in a closed position, allowing each of the ferrets to be isolated. Environmental enrichment was provided both in the cages (blankets, hanging cubes, and hammocks) and during 60 minute free exercise periods daily on weekdays. Ferrets were given *ad libitum* access to tap water and Totally Ferret Complete diet (Performance Foods, Broomfield, CO) with the exception of food restriction periods during training and testing.

During food restriction periods, ferret body masses were recorded every weekday and health was assessed daily (e.g., grooming, activity, visible signs of discomfort). There was no food restriction on weekends and ferrets were assessed for health daily by Animal Care staff. Food was provided after training or testing sessions for 1 hour while the ferrets were separated on different levels of the cages. Food bowls were weighed before and after the feeding session and the difference (i.e., mass of food assumed ingested) was recorded. Food and/or water restriction has been shown to be required for reliable operant conditioning responses in rats and minimally stressful in terms of behavior, appearance and physiology [14, 15]. Ferrets have also been shown to respond to operant conditioning tasks reliably with food restriction with little or no signs of stress [13].

### Stimuli—Duck feces

#### Cohort I—National Wildlife Research Center samples

Sixty mixed sex hatchery-bred mallards (Murray MacMurray, Webster City, IA, USA) were randomly assigned to one of four treatment groups of 15 ducks each. Fresh water and food were provided *ad libitum*. Feed consisted of game bird chow and cracked corn mixture (mixed 2:1). All birds were maintained in containment prior to experimentation and were screened for antibodies to IAV prior to inoculation to ensure they were negative. Testing occurred in BSL-2 containment rooms. All ducks were inoculated oro-choanally with 1 ml of BA-1 viral transport media (M199-Hank’s salts, 1% bovine serum albumin, 350 mg/L sodium bicarbonate, 2.5 mg/ml amphotericin B in 0.05 M Tris, 100 units/ml penicillin, 100 mg/ml streptomycin, pH 7.6) containing 0, 3, 4 or 5 log_10_ EID_50_ of A/environment/Illinois/NWRC183983-24/2006 (H6N2, GenBank CY122500.1). H5 (next cohort) and H6 viruses were used in these experiments because those subtypes are of high interest due to the increased likelihood that those subtypes spillover into poultry and cause economic harm. While H3s and H4s are the most commonly detected subtypes in N American waterfowl and can spillover into poultry, H3s and H4s do not generally cause pathogenicity in wild birds or poultry. Mallards were inspected daily for signs of pain or distress. Feces were collected from individual ducks daily on days 0, 1, 2, 3, 4, 7, 10, and 14 post-inoculation stored at -80°C until testing. All ducks were also swabbed and tested for viral RNA per qPCR methods described in [16] which confirmed infection in all inoculated individuals.

#### Cohort 2—Colorado State University samples

Ten farm-raised mallards of mixed sex were housed indoors (10 birds per pen). Fresh water and food were provided daily. Feed consisted of approximately100g of game bird chow and cracked corn mixture (mixed 2:1) per duck per day. All birds were maintained in containment prior to experimentation and were screened for antibodies to IAV prior to inoculation to ensure they were negative. Six ducks were infected ocularly, intranasally and orally with 1 ml of brain heart infusion broth containing 1.6 x 10^6^ plaque-forming units (pfu) of A/Mallard/MN/346250/00 (H5N2). Cloacal swabs were collected on days three and four following experimental infection. All individuals were inspected daily for signs of pain, distress, or infection. Infection was confirmed by real-time RT-PCR and inoculation into 10-day old embryonating chicken eggs. Two pooled fecal samples were collected from each duck. Feces were collected daily for four days immediately preceding experimental infection and again on days 1, 3, 5, 7, 9, 11, and 13 post-inoculation. Pre- and post-treatment samples were stored frozen at -80°C until training and testing.

Twenty farm-raised mallards of mixed sex were housed indoors (10 birds per biocontainment room). Housing, water, and food were provided consistent with LPAIV infected mallards. Ten ducks were infected by intratracheal inoculation of 0.15 ml containing 50,000 tissue culture infectious doses 50% (TCID_50_) of a lentogenic, field isolate of Newcastle disease virus (NDV). At the same time, another 10 ducks in a different room were inoculated with 20,000 TCID_50_ of a field isolate of infectious laryngotracheitis virus (ILV). For birds inoculated with either virus, cloacal swabs and feces were collected prior to virus inoculation and daily from days 1–5 following virus inoculation. Ten ducks were not inoculated and used as controls. Infection was confirmed by virus specific plaque assays of tracheal swab samples.

### Ferret odor alert response and odor discrimination training

**Fig 1:**
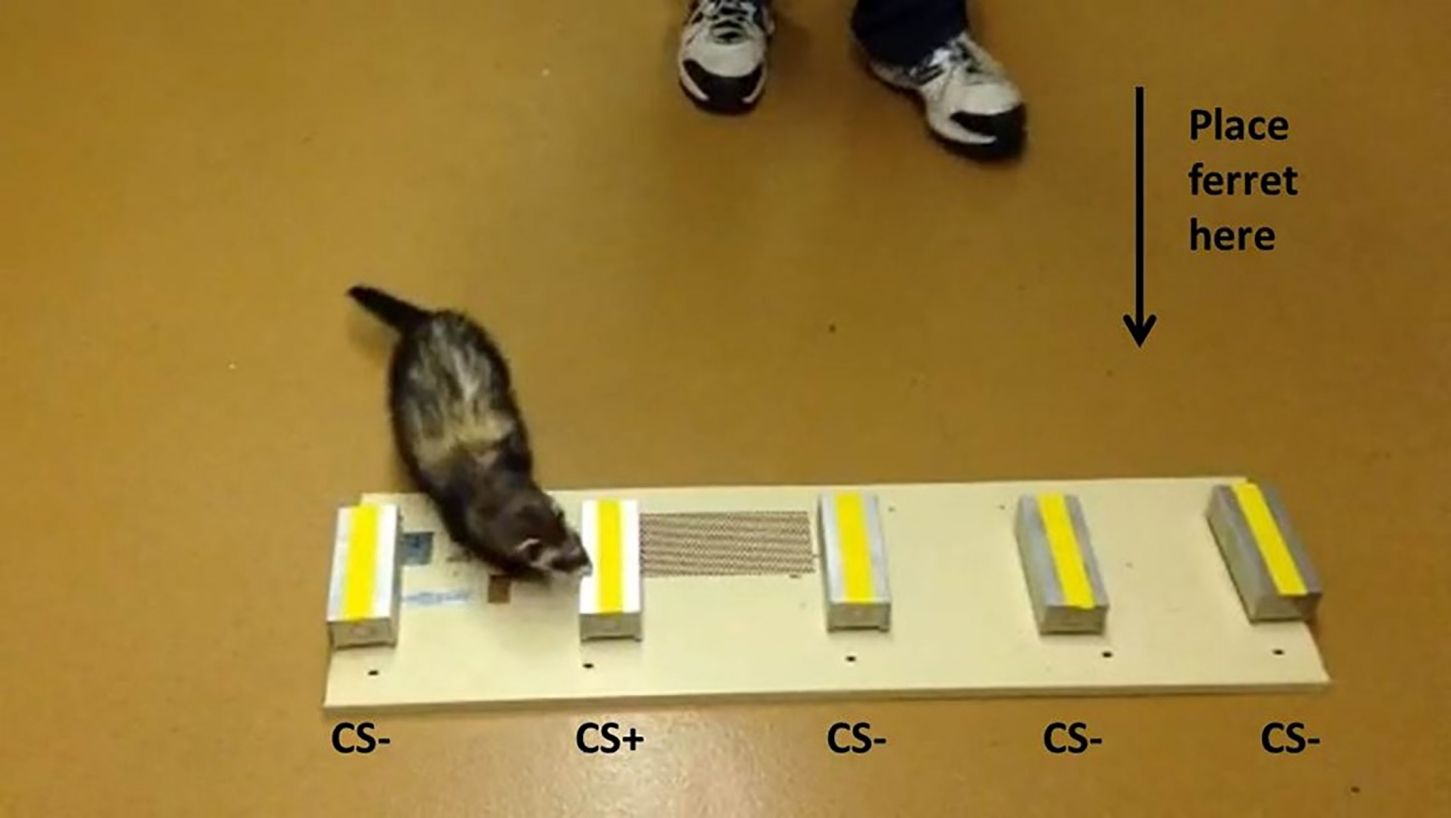
Scratch box panel used to monitor operantly conditioned responses of trained ferrets to odors emitted from fecal samples derived from LPAIV infected and non-infected donor mallards.

Ferrets were trained using 1 in 5 bioassays. Five scratch boxes were attached to a metal panel approximately 15.9 cm apart and in the longitudinal center of the board (Fig 1). The base compartment of each scratch box was customized to allow for the retention of a small 1 ml glass vial (Qorpak, Bridgeville, PA, USA). Vial caps (plastic septum-type screw caps with a 9 mm diameter opening) were fitted with 10 mm, Whatman qualitative filter paper, grade 1 (Sigma-Aldrich, USA) that allowed for the escape of volatiles but not the material placed in the vial (0.25–0.5 g of feces per vial). One randomly positioned box of the five on the panel held a vial containing fecal material from an LPAIV infected duck. The remaining four boxes held vials containing fecal material from non-infected ducks. Feces from an LPAIV infected duck were considered the conditioned stimulus positive (CS+) as the ferrets were rewarded for alerting to it and feces from non-infected ducks were considered the conditioned stimulus negative (CS-).

A session consisted of 12 trials for each of the six ferrets with the position of each box being pseudo-randomized for each trial. During training and subsequent testing, a “correct” selection by the ferret is the sample collected from an LPAIV infected animal. When an individual ferret correctly alerted to the box containing the CS+ sample, a clicker was activated, and the ferret was rewarded with a small amount of FerretVite with a modified syringe. It is important to note that 20% was the level chance of detecting the CS+ box as only 1 of 5 boxes contained a rewarded sample.

### Double-blind procedure

To avoid the possibility of the handler inadvertently communicating the position of the CS+ to the ferret, all sessions were conducted using a double-blind procedure. The coordinator positioned the CS+ and CS- scratch boxes, placed the board on the ground to signal the start of a trial, confirmed or rejected the ferret handler’s call (described in the next sentence), and picked up the board to position the boxes for the next trial. The ferret handler controlled when the ferrets were to start a trial, called out when a ferret alerted to one of the boxes, rewarded the ferret if the coordinator confirmed the choice (i.e., clicked the clicker and provided a small amount of FerretVite with a modified syringe), or picked up the ferret and walked away if the coordinator called the choice incorrect, and then faced away from the coordinator while the board was prepared for the next trial. This method was used for all trials that included the shaping of behavior, training, and experimental testing, specifically during rewarded trials but not during unrewarded trials.

A single daily session consisted of 12 trials. During initial training all correct selections were rewarded in all 12 trials. Once all ferrets demonstrated 75% accuracy in rewarded training trials, four extinction (training) or four generalization (testing) trials were introduced into each session (i.e., four non-rewarded extinction or generalization trials and 8 rewarded trials). The numbers of rewarded/unrewarded trials were determined experimentally in a previous study (PONE-D-20-11704R1) and based on instrumental learning theory. Extinction trials were no different than training trials except that the ferrets were not rewarded for correct selections. Whereas the stimuli presented during extinction trials were the same fecal samples that are presented in rewarded training trials, unrewarded generalization trials consisted of novel (a condition or design element that the ferrets had not previously experienced) stimuli. Because the ferrets experienced a neutral response from the handler immediately following an extinction or a generalization trial, we assumed that little or no learning occurred during these trials. An overview of the training and testing that used these methods can be found in Table 1.

**Table 1:** An overview of the training and experiments.

Experiment	Sample Cohort	Description
**Training**	1	Having been trained to irradiated duck fecal samples, ferrets were trained and tested on duck fecal samples that remained active.
**1**	1	To determine if ferrets could generalize the odor profile of infection status learned in training, ferrets were tested with novel fecal samples from AIV infected and non-infected ducks.
**2a**	1	To determine if ferrets were using the age of the fecal sample rather than the odor profile associated with infection status, ferrets were presented with a panel of samples that were collected on the same day with the exception of 1 fecal sample collected from a non-infected duck on a different day (see Table 2).
**2b**	1	To determine if ferrets were using the age of the fecal sample rather than the odor profile associated with infection status, ferrets were asked to discriminate between dual positive samples. Each panel contained varying collection day combinations (see Tables 3 and 4).
**3a**	1 and 2	To determine if ferrets could discriminate between completely novel samples from cohort 2 that included pre and post infection samples from the same individuals but differed from cohort 1 with respect to duck identity, housing, feed, and duration of sample storage prior to training.
**3b**	1 and 2	This experiment was conducted to determine if ferrets trained to detect an odor representative of LPAIV infection would generalize to fecal samples collected from ducks infected with a different virus (See Table 5).

### Demonstration of learned response

To train ferrets to respond to fecal samples from novel individual ducks and inoculation doses for mallards infected with LPAIV, rewarded trial CS+ samples consisted of fecal samples from novel individual ducks from the 3 or 5 log_10_ EID_50_ H6N2 inoculation dose groups representing all collection days (i.e., collection days 2–14 post inoculation) where real time RT-PCR revealed ongoing viral shedding. We assumed these were the optimal sample days to use where there should be ongoing odor signals the ferrets were likely using as cues that were due to viral induced metabolic changes.

CS- samples consisted of samples from randomly chosen novel uninfected control individual ducks representing all collection days (days 0–14). Following two days of all rewarded training trials, we introduced unrewarded trials into the sessions. One session group that included four days of extinction trial sessions (n = 96) and a second session group that included three days of extinction trial sessions (n = 72). In the second session group, extinction trial CS+ samples consisted of samples from familiar ducks representing novel collection days (days 2–14) for that individual (fresh fecal samples). That is, CS+ samples used in the extinction trials were from the same ducks, but different collection days than previously used.

Each daily session included four extinction trials and eight rewarded trials per ferret randomly presented during twelve trials per session. The same fecal samples were used across all four days. After four days, the ferrets were performing at 85% or better and were able to move onto the next stage of testing with further extinction trials. The next three days of testing sessions used fresh samples from the same ducks, but novel collection days and consisted of 72 overall unrewarded extinction trials across the 6 ferrets, again with four extinction trials per ferret per day. After three days of this level of training, ferrets were performing at 85% or better. The performance at the end of these training trials indicated the ferrets were ready for testing sessions with novel samples (i.e., novel individual ducks) presented in unrewarded generalization trials.

### Experiment 1—Discrimination of feces from LPAIV infected ducks

The ferrets’ response to samples collected from novel ducks was examined in unrewarded generalization trials interspersed among rewarded trials. Rewarded trial CS+ samples consisted of randomly chosen novel individual ducks inoculated with the 3 or 5 log_10_ EID_50_ doses representing collection days 2–14. Generalization trial CS+ samples consisted of novel individual ducks inoculated with the 4 log_10_ EID_50_ dose and representing collection days 2–14. Generalization CS- samples consisted of control ducks representing collection days 1–14 and collection day 0 samples from infected ducks.

We again ran two testing sessions, one that included three days of generalization trial sessions and a second that included four days of generalization trial sessions. It was apparent after the first three days of testing that the collection day 0 from mallards inoculated at 4 log_10_ EID_50_ were being selected as CS+ at rates higher than anticipated. Despite a relatively high-performance mean accuracy of 81%, during these trials, we noticed that individual ferrets making the most errors were choosing the fecal samples from specific individual donors (mallards inoculated with 4 log_10_ EID_50_). We removed from testing and training all day 0 fecal samples from these specific donors because of these confounding effects (see Discussion). We then began the next four days of testing. Four days of running these reconfigured panels included 96 unrewarded generalization trials. Each daily session included four generalization trials presented during twelve trials per session. Each generalization trial consisted of one of 10 novel individual ducks inoculated at the 4 log_10_ EID_50_ dose representing sample collection across days 1–14.

### Experiment 2a—Discrimination based on collection day

To examine if the time since infection for collection of fecal samples was utilized in making a correct choice in lieu of infection odor identity, ferrets were presented with novel duck fecal samples in unrewarded generalization trials consisting of a CS+ and three of four CS- samples coming from the identical collection day. The fourth CS- sample was from a differing collection day on the opposite end of the collection spectrum from the other four samples. For example, if the panel consisted of four collection day 3 samples, the remaining CS- sample would be from collection day 7, 14, or 10).

Rewarded trial CS- and CS+ samples consisted of randomly chosen control or infected fecal samples from individual ducks representing collection days 3–14. Generalization trial CS- samples consisted of samples from control ducks with 3 sample boxes representing collection days that matched the collection day of the CS+ (Table 2). The fourth CS- sample box contained a sample from a completely different collection day. Three days of running these panel configurations included 72 unrewarded generalization trials.

**Table 2:** Generalization panel configuration for odd CS- collection day testing.

Session day	CS- box 1	CS- box 2	CS- box 3	CS- box 4 odd collection day	CS+ box
**1**	CD 3	CD 3	CD 3	CD 1, 2, 10, or 14	CD 3
**2**	CD 4	CD 4	CD 4	CD 1, 2, 10, or 14	CD 4
**3**	CD 7	CD 7	CD 7	CD 1, 2, 10, or 14	CD 7

The order of the boxes in each panel were not presented as shown above. The boxes were presented to in random order for each ferret. Collection day (CD).

### Experiment 2b—Discrimination with dual CS+ samples

In order to further challenge the hypothesis that ferrets were merely choosing the sample that differed the most from the other samples, two CS+ samples from different individual ducks and with differing collection days from each other and from the CS- samples were included in the panel configurations for the generalization trials. All rewarded trial samples were from collection days 4–14 with CS- samples from control ducks and CS+ samples from infected ducks (any inoculation dose group). Generalization CS- samples consisted of control duck samples from the same collection day. Generalization CS+ samples were from two individual infected ducks from the same or differing collection days (Table 3). This portion of the experiment utilized a single session for each ferret.

**Table 3:** Generalization panel configuration for discrimination with dual CS+ samples collection day testing.

Generalization trial number	CS- box 1	CS- box 2	CS- box 3	CS+ box 1	CS+ box 2
**1**	CD 3	CD 3	CD 3	CD 7	CD 10
**2**	CD 3	CD 3	CD 3	CD 7	CD 14
**3**	CD 3	CD 3	CD 3	CD 3	CD 10
**4**	CD 3	CD 3	CD 3	CD 3	CD 3

The order of the boxes in each panel were not presented as shown above. The boxes were presented to in random order for each ferret.

Additional generalization trials were conducted in which the 3 CS- samples and a CS+ sample presented were all from the same collection day and a second CS+ sample was from a differing collection day. Thus, only the odd CS+ had a different collection day in comparison to the remaining samples with the exception of trials with all the same collection day (Table 4). Rewarded trial CS- and CS+ (any inoculation dose) samples were chosen across individual ducks from collection days 4–14. This portion of the experiment was a single session for each ferret.

**Table 4:** Generalization panel configuration for discrimination with dual CS+ samples collection day testing.

Generalization trial number	CS- box 1	CS- box 2	CS- box 3	CS+ box 1	CS+ box 2
**1**	CD 10	CD 10	CD 10	CD 10	CD 1
**2**	CD 10	CD 10	CD 10	CD 10	CD 4
**3**	CD 10	CD 10	CD 10	CD 10	CD 10
**4**	CD 10	CD 10	CD 10	CD 10	CD 14

The order of the boxes in each panel were not presented as shown above. The boxes were presented to in random order for each ferret. Collection day (CD).

### Experiment 3a—Discrimination of novel live virus samples

We collected pre and post infection samples from a new cohort (cohort 2) of mallards reared and subsequently infected at CSU for use in unrewarded generalization trials. Feces from cohort 1 were used in rewarded trials. We ran these panel configurations for two days, resulting in 48 unrewarded generalization trials across the six ferrets.

### Experiment 3b—LPAIV specificity testing

To determine if ferrets trained to detect an odor representative of LPAIV infection would generalize to fecal samples collected from ducks infected with a different virus, we conducted generalization trials with one control fecal sample from each duck cohort, a post NDV infection sample, a post ILTV infection sample, and one CS+ sample from a LPAI-infected duck (Table 5) after a single day of reward training with cohort 1 samples. NDV and ILTV were chosen because infection by these pathogens result in respiratory/gastrointestinal effects and similar associated clinical manifestations (i.e., sneezing, coughing, diarrhea, and weight loss) in poultry. We ran specificity panels for three days which resulted in 72 unrewarded generalization trials.

**Table 5:** Panel configuration for specificity testing.

	Box 1	Box 2	Box 3	Box 1	Box 2
**Rewarded trials**	CS-	CS-	CS-	CS-	CS+
	Cohort 1	Cohort 1	Cohort 1	Cohort 1	Cohort 1
	CD 3–14	CD 3–14	CD 3–14	CD 3–14	CD 3–14
**Generalization trials**	CS-	CS-	NDV	ILV	LPAIV
	Cohort 1	Cohort 2	Cohort 2	Cohort 2	Cohort 2
	CD 3–14	Pre-infection	CD 3–11	CD 3–11	CD 3–11

The order of the boxes in each panel were not presented as shown above. The boxes were presented to in random order for each ferret. Collection day (CD). Newcastle disease virus (NDV). Infectious laryngotracheitis virus (ILV). Low pathogenic avian influenza virus (LPAIV).

### Data analysis

Cumulative responses across all trained ferret trials were calculated for each set of experimental generalization trials. Success rates (number of correct trials divided by the total number of generalization trials) were statistically evaluated using binomial proportion tests with a continuity correction for small numbers of observations [17]. The data were tested for independence between donor identity, testing day, and correct responses using the Cochran-Mantel-Haenszel test [18]. Day 0 fecal sample trials were not included in analysis as the actual day of infection could not be confirmed. Experiment 2b data (two CS+ samples in a panel) were not statistically analyzed, but we report the total accuracy percentage for choosing a CS+. For all other experiments, a success rate of 20% would be expected by chance. However, as the goal of these trials was to demonstrate the high specificity of trained biosensors, ferret responses were also compared to 50% and 75% success rates.

## Results

### Demonstration of learned response

**Fig 2:**
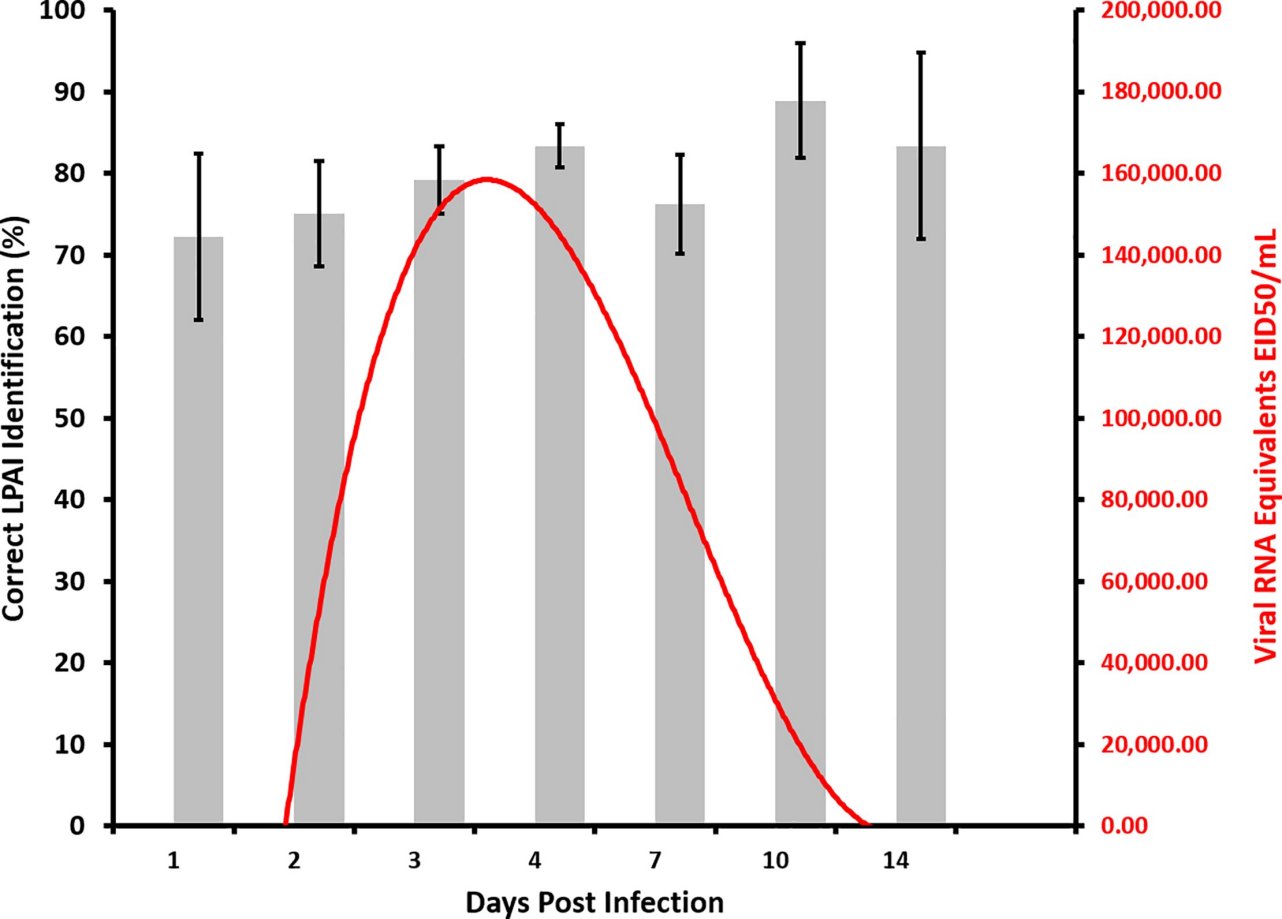
Ferrets are accurate in detecting fecal samples from infected ducks exposed to LPAIV well before and after peak viral shedding as confirmed by qPCR. During training and testing, ferrets correctly (grey bars; left y axis) identified the location of a single fecal sample (n = 480; Cohort 1; all inoculation doses) from a LPAIV infected duck presented among four negative samples with greater than 20% accuracy (chance) regardless of the number of days post infection. Calibrated qPCR results of fecal samples from ducks inoculated with LPAIV (red line; right y axis) show virus shedding starts on day 2 post-infection and climbs steeply to a peak on post-infection day 4 and slowly declines to zero on 14 days post-infection.

The first four day extinction trial sessions were conducted to determine if the performance of the ferrets was accurate enough to move on to testing. The first day, the accuracy performance of individual ferrets ranged from 67–92% (i.e., 67, 83, 92, 92, 92, and 92). By day 2, the ferrets were performing at 93% accuracy with individual ferret performances ranging from 92–100%. By day 4, the ferrets were still performing at 93% accuracy overall with individual ferret performances ranging from 75–100%. The next three days of double-blind testing used fresh samples from familiar individual duck donors, but novel collection days, resulted in 72 overall unrewarded generalization trials. The first day, the accuracy performance of individual ferrets ranged from 75–92%. By day 3, the ferrets were performing at 96% accuracy with individual ferret performances ranging from 83–100%. Interestingly, when the ferret response to positive samples is compared to viral RNA equivalents EID50/mL as confirmed by calibrated qPCR [16], the ferrets were still highly accurate in alerting to the positive sample for several days after viral shed was greatly diminished (Fig 2).

### Experiment 1—Discrimination of feces from LPAIV infected ducks

**Fig 3:**
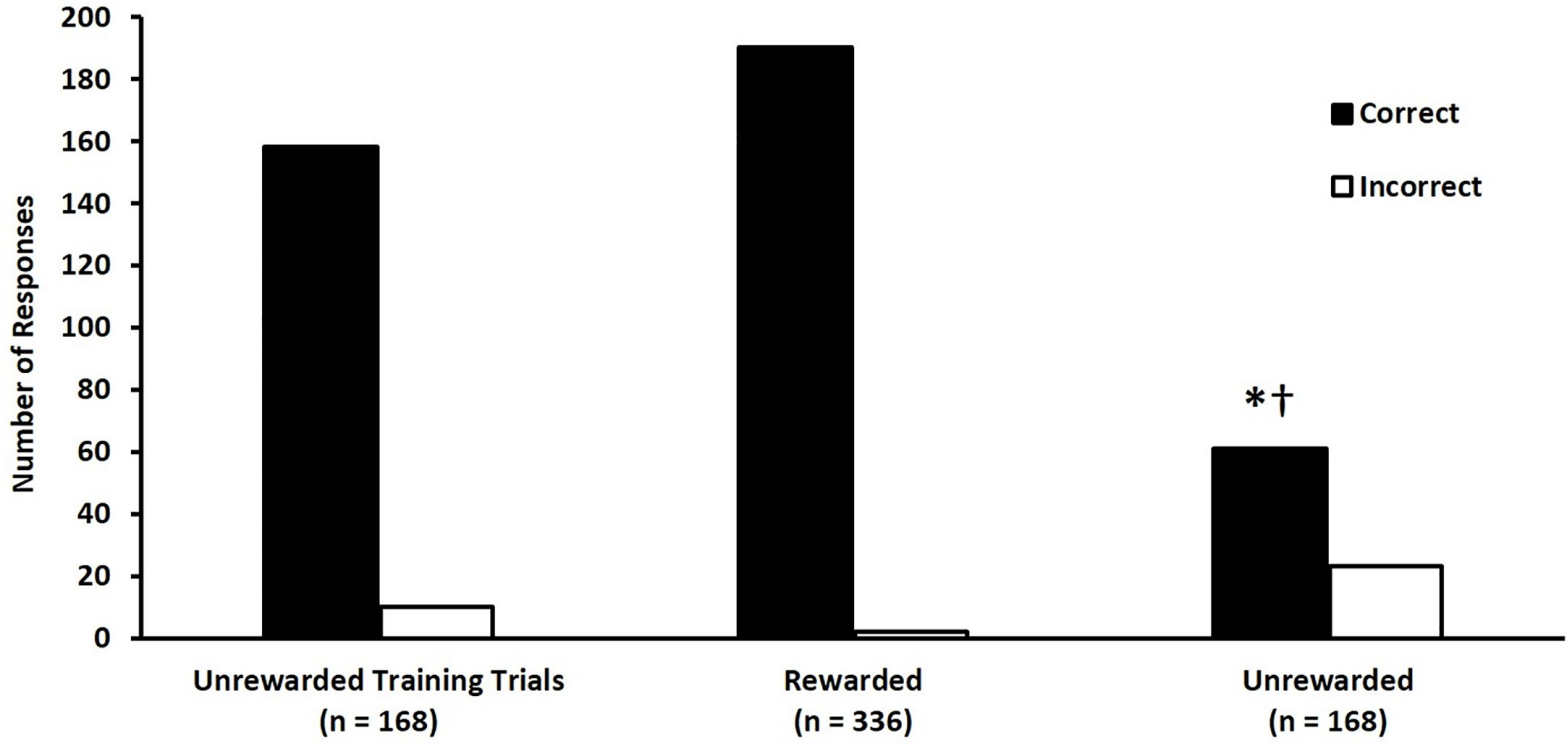
Ferrets are capable of generalizing their ability to discriminate between control fecal samples and infected fecal samples from ducks exposed to low pathogenic avian influenza A virus (LPAIV) when they encounter fecal samples from novel individuals. During training, ferrets correctly (black bars) identified the location of a single fecal sample from a LPAIV infected duck presented among four negative samples with 94% accuracy. During rewarded testing trials, ferrets correctly identified the location of infected samples with 99% accuracy. During unrewarded generalization trials, ferrets correctly identified infected samples from novel ducks from uninfected samples with 73% accuracy, which was significantly greater than null hypotheses of 20% (*p < 0.0001) and 50% (†p < 0.0001), but not statistically different from 75% (p = 0.3071). White bars represent incorrect choices.

Trained ferrets were highly accurate (94% correct choices in unrewarded training trials over 4 testing days) at discriminating between fecal samples collected from control mallards and from mallards across the entire spectrum of collection days (Fig 3, left two bars).

All six trained ferrets correctly identified the location of the single CS+ sample derived from an infected donor with 99% accuracy (Fig 3, middle bars) across four days of rewarded testing trials. During unrewarded generalization trials, ferrets correctly identified the location of the single sample derived from an infected donor with 73% accuracy (Fig 3, right two bars). This result is statistically different from chance (20%; p < 0.0001) and a 50% success rate (p < 0.0001) and is not statistically different from a 75% success rate (p = 0.31). Non-parametric analysis indicated that neither test day nor donor identity were associated with the selection made by the ferrets in either rewarded testing (p = 0.55) or unrewarded generalization trials (p = 0.48).

### Experiment 2a—Discrimination based on collection day

After Experiment 1 confirmed the trained ferrets were highly accurate and reliable in their ability to discriminate between fecal samples collected from control mallards and LPAIV infected mallards there was no further attempt at training. To determine if the time since infection was utilized in making a correct choice in lieu of infection odor identity, we challenged the ferrets by offering a panel where one CS- sample was from a different collection day in comparison to the remainder of the panel or offering a panel where there were two potentially correct choices, but only one of the two choices represented an odd collection date.

**Fig 4:**
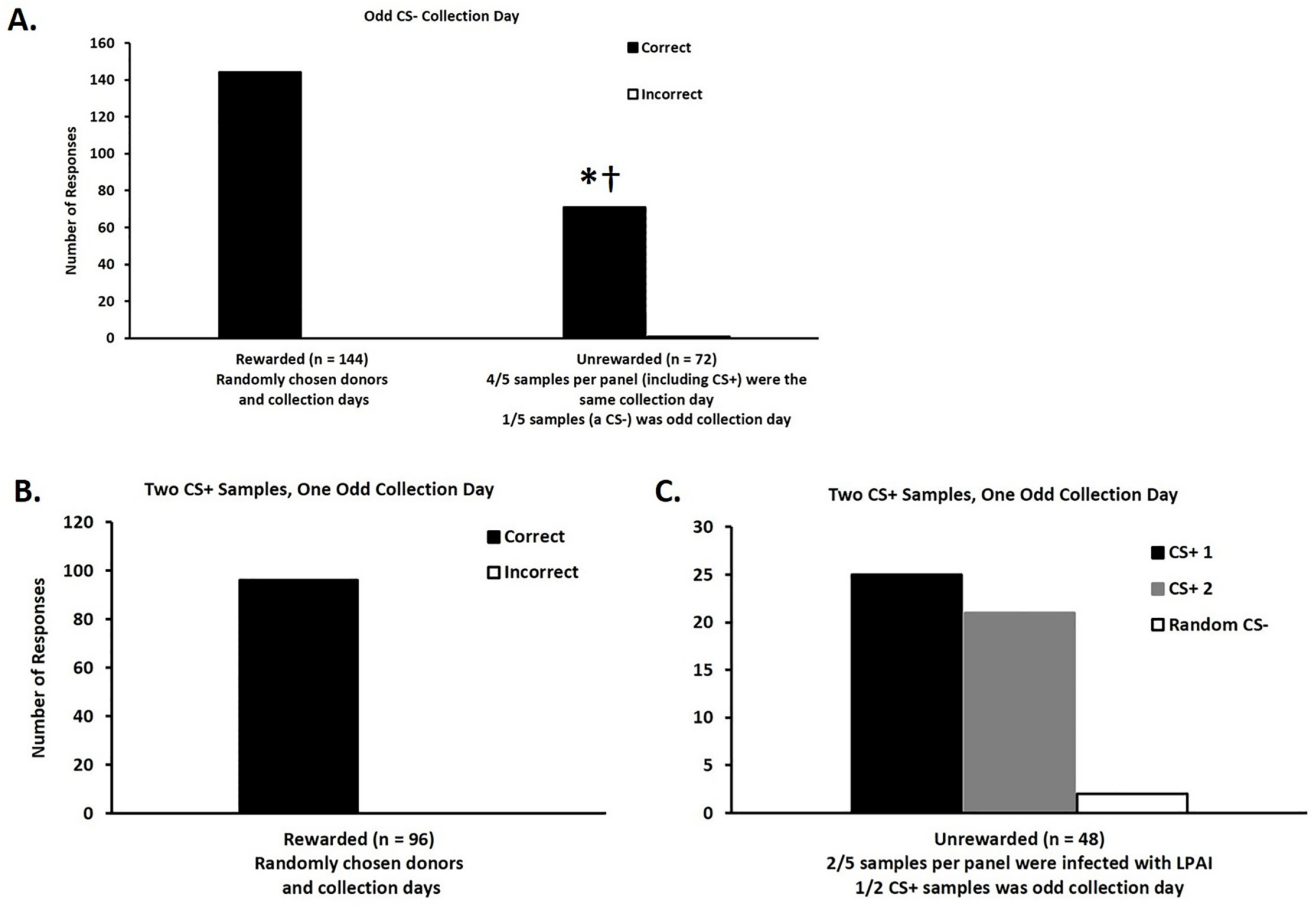
Ferrets are capable of identifying novel fecal samples from LPAIV infected ducks based on an odor change derived from infection and not based on odor changes derived from the time since infection. A) During 144 rewarded testing trials, ferrets correctly (black bars) identified the location of the single LPAIV post-infection fecal sample with 100% accuracy. During the 72 unrewarded generalization trials, ferrets correctly identified the location of the single post-infection fecal sample with 99% accuracy (*p < 0.0001) rather than negative samples from a different collection day. These classification rates are significantly greater than the null hypotheses of 20%, 50%, and 75% success rates (†p < 0.0001). White bars represent incorrect choices. B) During 96 rewarded testing trials, ferrets correctly identified the location of the post-infection samples with 100% accuracy. C) During 48 unrewarded generalization trials, ferrets correctly identified the CS+ samples with 96% accuracy (52% accuracy for identical CD and 44% accuracy for the odd CD). White bars represent incorrect choices.

All six trained ferrets correctly identified the location of a post-infection sample with 100% accuracy (Fig 4A) over three days of rewarded trials even when challenged with a negative sample from a different collection day. A score of 100% during these trials meant there was no need to examine the roles of ferret or session on successful identification of the CS+. During unrewarded generalization trials, ferrets correctly identified the location of the post-infection sample with 98% accuracy (Fig 4A) when the panel included a CS- sample from an odd collection day. This result is statistically different from chance (20%), and 50% and 75% success rates (p < 0.0001). Non-parametric analysis of the data indicated that neither test day nor donor identity were associated with selections made in these unrewarded generalization trials (p = 0.42).

### Experiment 2b—Discrimination with dual CS+ samples

All six trained ferrets correctly identified the location of the single sample derived from an infected donor with 100% accuracy (Fig 4B) across two days of rewarded trials during testing. During unrewarded generalization trials, ferrets correctly identified the location of one of the two samples in the panel derived from an infected donor with 96% accuracy (52% accuracy for identical CD and 44% accuracy for the odd CD; Fig 4C).

### Experiment 3a—Discrimination of novel live virus samples

This experiment was designed to determine if trained LPAIV detection ferrets could discriminate between completely novel samples from cohort 2 that included pre and post infection samples from the same individuals but differed from cohort 1 with respect to duck identity, housing, feed, and duration of sample storage prior to training.

**Fig 5:**
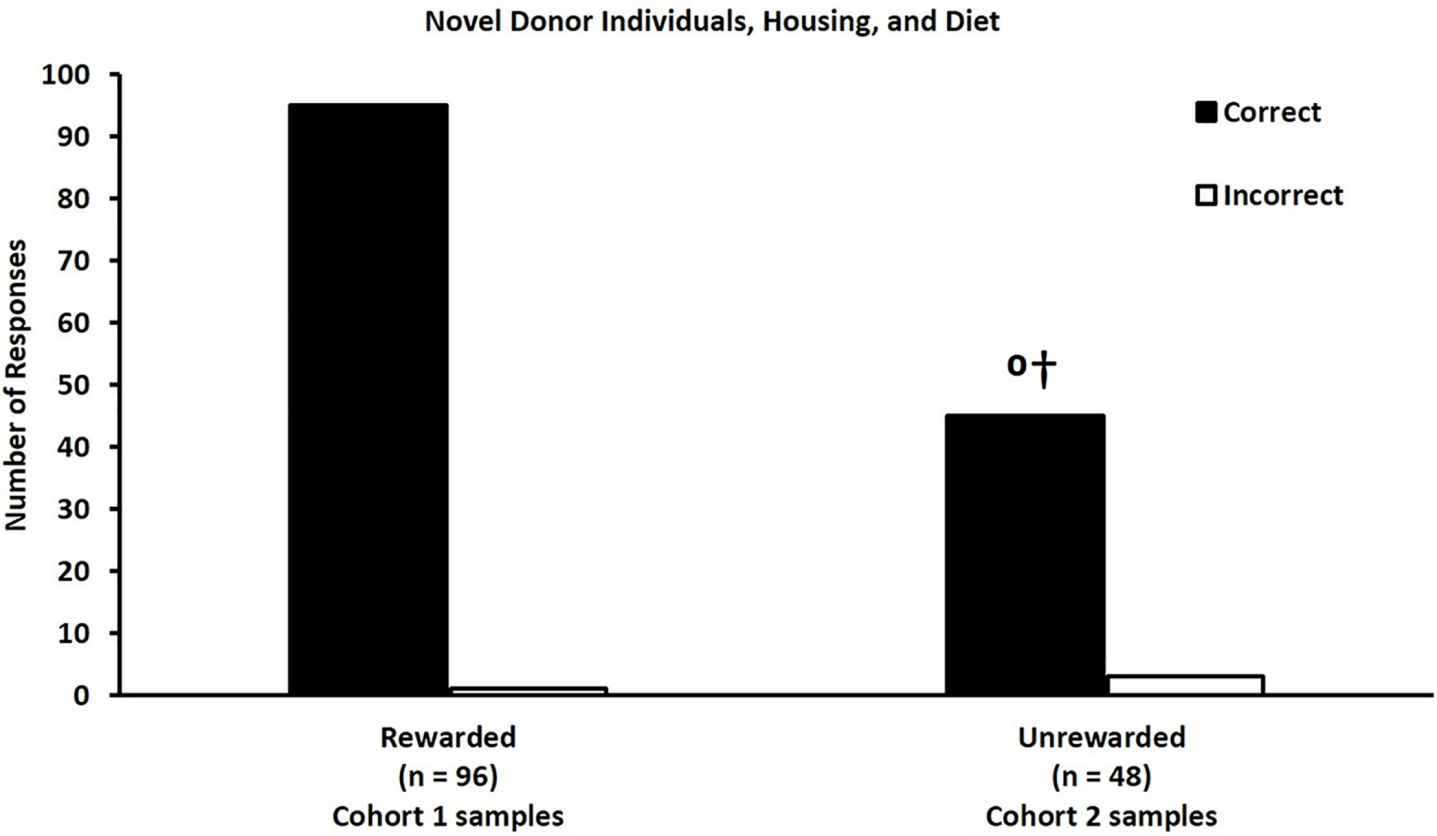
Ferrets are capable of identifying novel fecal samples from LPAIV infected ducks based on an odor change derived from infection and not based on an odor change derived from duck identity, housing, and diet. During rewarded testing trials, ferrets correctly (black bars) identified the location of the post-infection samples with 99% accuracy. During unrewarded generalization trials, ferrets correctly identified the novel positive samples with 94% accuracy, which was significantly greater than the null hypotheses of 20%, 50% (⁰p < 0.0001), and 75% (†p < 0.0013) success rates. White bars represent incorrect choices.

All six trained ferrets correctly identified the location of the single positive sample with 99% accuracy (Fig 5) across two days of testing. During unrewarded generalization testing, ferrets correctly identified the location of the single positive sample derived from a novel infected donor with 94% accuracy (Fig 5). This result is statistically different from chance (20%), and 50% (p < 0.0001) and 75% (p < 0.0013) success rates. Non-parametric analyses for the rewarded and unrewarded trials indicated that neither test day nor donor identity were associated with the selection made by the ferrets in rewarded testing trials (p = 0.42) or unrewarded testing trials (p = 0.69).

### Experiment 3b—LPAIV specificity testing

To determine if ferrets identified the location of the single sample derived from an infected donor based on a pathogen specific infection odor, ferrets trained to detect an odor representative of LPAIV infection were tested with a panel that included fecal samples negative for LPAIV infection but collected from ducks infected with NDV and/or ILTV in addition to non-infected controls.

**Fig 6:**
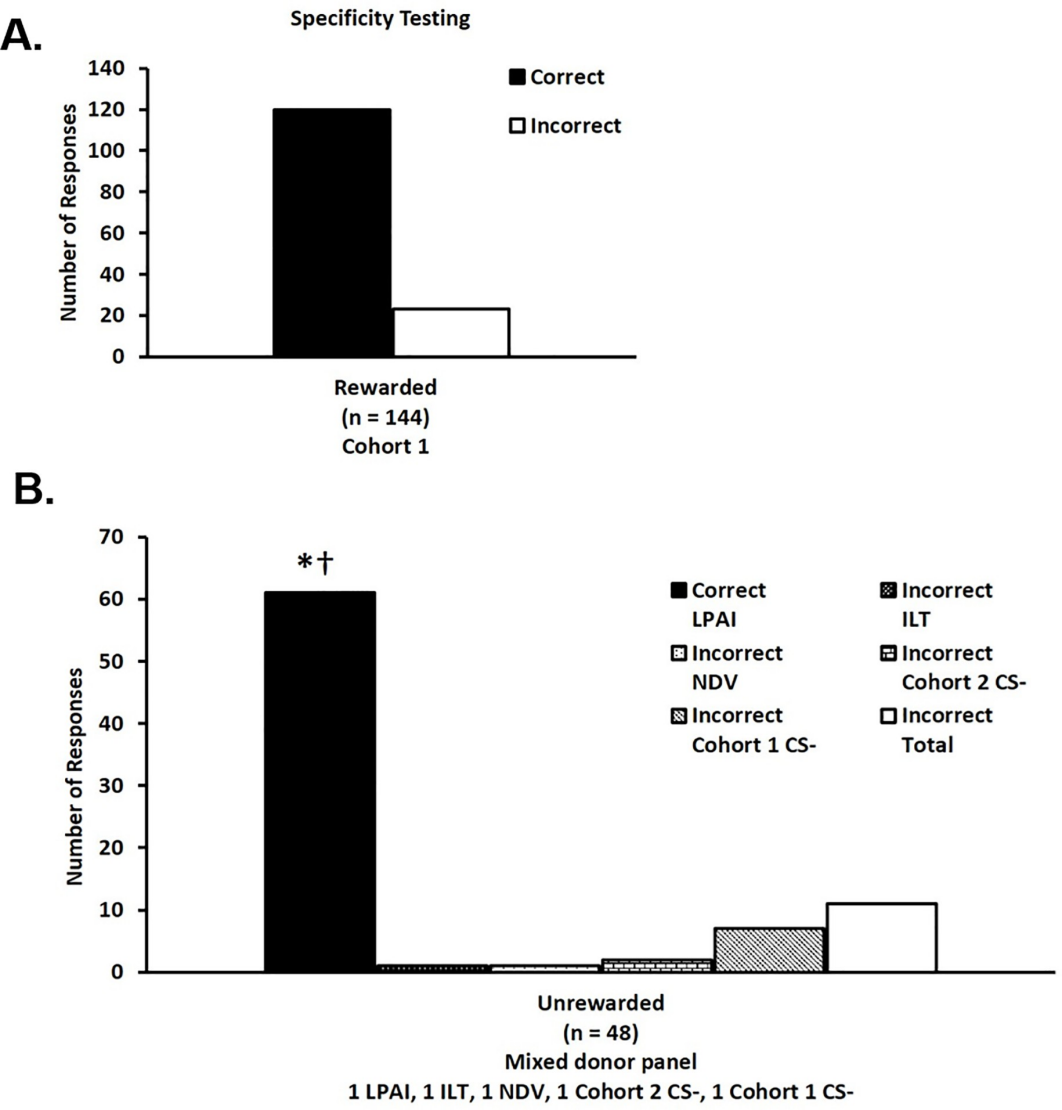
The results from experimental trials including samples from ducks infected with multiple pathogens suggest the odor cue used by ferrets to detect avian influenza is specific to AIV infection and is not a general immune response resulting from infection. A) During 143 rewarded testing trials, ferrets correctly (black bars) identified the location of the positive samples with 84% accuracy. B) During 48 unrewarded generalization trials, ferrets correctly identified the novel LPAIV samples with 85% accuracy, which was significantly greater than the null hypotheses of 20%, 50% (⁰p < 0.0001), and 75% success rates (†p < 0.028). Dotted black bars represent incorrect choices for samples from ducks infected with infectious laryngotracheitis virus (ILTV). Dotted white bars represent incorrect choices for samples from ducks infected with Newcastle disease virus (NDV). Brick white bars represent incorrect choices for samples collected in cohort 2 from non-infected ducks. Horizontal stripe white bars represent incorrect choices for samples collected in cohort 1 from non-infected ducks. White bars represent total incorrect choices.

All six trained ferrets correctly identified the location of the single positive sample derived from an LPAIV infected duck with 84% accuracy (Fig 6A) across three days of testing. During unrewarded generalization testing, ferrets correctly identified the location of the single positive sample from a LPAIV infected donor with 85% accuracy (Fig 6B). This result is statistically different from chance (20%), 50% (p < 0.0001) and 75% (p < 0.0284). Non-parametric analysis of the data indicated that both test day and donor identity were associated with the selections made by the ferrets in rewarded testing trials (p = 0.03) but there was no association in unrewarded testing trials (p = 0.64).

## Discussion

The results of this set of experiments provide clear-cut evidence that ferrets are not only capable of performing an olfactory learning discrimination task but can also be utilized to perform non-invasive detection of a change in the health status of LPAIV infected mallards. Although different assays, these results confirm the results of a previous experiment on the ability of a biodetector to detect the presence of LPAIV infection at a high rate of accuracy: mice trained as biodetectors demonstrated better than 80% accuracy in choosing between a pair of odors by choosing a y-maze arm associated with fecal odors from infected ducks [9]. In the present study, ferrets demonstrated better than 90% accuracy for detecting novel samples from AIV infected ducks in a 1-in-5 assay (Fig 5).

However, there did seem to be a learning curve in the detection of samples from novel donors after training with samples from “familiar” mallards that the ferrets had previously and repeatedly experienced (Fig 3). These results suggest that either the strategy and odor identity utilized by the ferrets in choosing the samples donated by infected individuals morphs with each trial dependent on the properties of the current samples or that several weeks of training were needed to cement the odor identity of LPAIV post-infection samples in the presence of potentially confounding variables such as date collected, familiarity, or concentration of inoculation dose. As the accuracy of the ferrets improved with repetition, it is likely the latter is the probable explanation.

In the final experiment, ferrets were offered a panel of two samples from healthy LPAIV negative mallards, one sample from a mallard infected with NDV, and one sample from a mallard infected with ILTV along with a single sample from a LPAIV infected mallard. This experiment was designed to evaluate the ability of trained animals to discriminate among fecal samples collected from mallards experimentally infected with other viruses having respiratory and/or gastrointestinal effects. If fecal odors differ according to infectious agent or their associated clinical manifestations, the ferrets were hypothesized to perform with a high rate of correct identification. The results (Fig 6B) suggest that the odor identity utilized by the ferrets to identify the box holding a fecal sample collected from an LPAIV infected donor is specific for avian influenza and is not comparable to infection with other viruses that result in the exhibition of similar respiratory/gastrointestinal effects. This is compatible with studies that suggest that cell lines infected with different viruses and even different AIV subtypes all produce unique volatile compounds that could be used as volatile olfactory markers [19].

Fecal matter from infected waterfowl contain viable avian influenza A virus and consequently, fecal sampling of wild waterfowl and their habitats is an integral part of any surveillance system designed for the early detection of emerging avian AIVs that pose a threat to human and livestock health. Fecal sampling in the wild is economically feasible, although the samples collected must be fresh and inevitably contain some form of environmental or alternate pathogen contamination [3]. For example, herbicides or land cover treatments are detrimental to samples (i.e. can kill virus). Our current results suggest that the use of biodetectors trained to detect and identify fecal matter derived from waterfowl infected with avian influenza A viruses would add a layer of surveillance screening to the current system that greatly decrease the cost of molecular diagnostics so the number of negative samples that need to be tested would be greatly decreased.

While ferrets provide a strong laboratory model, they would not provide the control and sensitivity required of an effective biodetector in the field. Ferrets can be easily distracted by novel odors, objects, and people. However, work completed with ferrets in the laboratory decreases the need for some experiments to be repeated and reveals some of the positive outcomes and potential pitfalls of working with mammalian biodetectors. For example, individual ferrets were not unique about their choice of several day 0 samples. Some of the day 0 samples were collected only hours after the ducks were inoculated but 4 or more ferrets alerted to these samples as being positive for infection. This is either a potential confound or the ferrets detected an odor change that is occurring rapidly after inoculation. This is supported by the data shown in Fig 1 where the ferrets appear to be performing at better than chance (i.e., 20%) on days 1 and 14. These days are before and after the virus was detectable by qPCR for the samples in this study (Fig 1). It would be interesting to examine how early the odor signal is detectable by mammalian olfaction and how long the signal persists. This is an issue that can be considered when designing experiments using canine biodetectors. Trained detector dogs have already been shown to be invaluable tools for wildlife field research. Dogs have been employed for scat [20–22], carcass [23], and pest detection [24]. Furthermore, the ability of dogs to “diagnose” certain human diseases has been demonstrated in a number of scientific studies, including lung, prostate, colorectal, ovarian, breast, bladder, and skin cancers or malaria [1, 25].

The current study provides evidence that biodetectors can be used to detect fecal samples from mallards infected with avian influenza A viruses. This evidence shows that ferrets can identify the signature odor that is a result of infection from LPAIV and that this odor identity is specific for LPAIV infection. This odor identity is not compromised by days since infection (provided that the sample is collected within 24 hours of deposit and is kept at -40⁰ or colder), exposure dosage, individual duck identity, or husbandry methods. Nonetheless, important questions remain to be answered. For example, can a biodetector detect the difference between fecal samples from waterfowl infected with HPAIV from LPAIV, or differentiate between samples that result from infection with different subtypes or strains of AIV? Most importantly, it will be interesting to determine whether a biodetector trained to identify LPAIV in mallard fecal samples and identify the odor identity of AIV in another species. This study clearly demonstrates the feasibility of deploying trained biodetectors for AIV surveillance in waterfowl.
